# Ablation of GSDMD Improves Outcome of Ischemic Stroke Through Blocking Canonical and Non-canonical Inflammasomes Dependent Pyroptosis in Microglia

**DOI:** 10.3389/fneur.2020.577927

**Published:** 2020-11-23

**Authors:** Kankai Wang, Zhezhe Sun, Junnan Ru, Simin Wang, Lijie Huang, Linhui Ruan, Xiao Lin, Kunlin Jin, Qichuan Zhuge, Su Yang

**Affiliations:** ^1^Zhejiang Provincial Key Laboratory of Aging and Neurological Disorder Research, The First Affiliated Hospital of Wenzhou Medical University, Wenzhou, China; ^2^Department of Neurosurgery, The First Affiliated Hospital of Wenzhou Medical University, Wenzhou, China; ^3^Department of Cerebrovascular, The Affiliated Hospital of Medical School of Ningbo University, Ningbo, China; ^4^Department of Pharmacology and Neuroscience, University of North Texas Health Science Center, Fort Worth, TX, United States

**Keywords:** ischemia/reperfusion injury, pyroptosis, neuroinflammation, gasdermin D, microglia, neurological outcome

## Abstract

Ischemia/reperfusion (I/R) injury is a significant cause of mortality and long-term disability worldwide. Recent evidence has proved that pyroptosis, a novel cell death form, contributes to inflammation-induced neuron death and neurological function impairment following ischemic stroke. Gasdermin D (GSDMD) is a newly discovered key molecule of cell pyroptosis, but its biological function and precise role in ischemic stroke are still unclear. The present study investigates the cleavage activity of GSDMD, localization of pyroptotic cells, and global neuroinflammation in *gsdmd*^−/−^ mice after I/R. The level of cell pyroptosis around the infarcted area was significantly increased in the acute phase of cerebral I/R injury. The ablation of GSDMD reduced the infraction volume and improved neurological function against cerebral I/R injury. Furthermore, we confirmed I/R injury induced cell pyroptosis mainly in microglia. Knockdown of GSDMD effectively inhibited the secretion of mature IL-1β and IL-18 from microglia cells but did not affect the expression of caspase-1/11 *in vitro* and *in vivo*. In summary, blocking GSDMD expression might serve as a potential therapeutic strategy for ischemic stroke.

## Introduction

According to systematic analysis studies of the global burden of disease (GBD) between 1990 and 2017, acute ischemic stroke has become the leading cause of mortality and disability worldwide (1–3), causing severe economic and healthy burdens. Intravenous thrombolysis and selective mechanical thrombectomy are the optimal therapeutic measures for ischemic stroke up till now (4, 5); however, the applicable proportion of intravenous thrombolysis only accounts for 3% of total ischemic stroke patients. Therefore, further research on the pathogenesis and interventions of ischemic stroke is urgently needed.

The primary injury of ischemic stroke is mainly caused by vascular occlusion which leads to neuron death and release of damage-associated molecular patterns (DAMPs) in focal ischemic tissue. A secondary immune response is subsequently induced in the injury area, characterized by activation of resident cells (mainly microglia), recruitment of peripheral cells (neutrophils, monocytes/macrophages, and other cells) (6, 7), and rapid induction of cascaded events, including the release of pro-inflammatory mediators, blood-brain barrier (BBB) damage, brain edema, and nerve cell death (6, 8). It is reasonable to speculate that blocking the release of inflammatory mediators may be beneficial for stroke recovery.

Pyroptosis, a newly discovered proinflammatory programmed cell death, has drawn increasing attention for its unique characteristics, such as cell swelling, bulging of the plasma membrane, secretion of inflammatory cytokines, and cell lysis (9). The pyroptotic process is dependent on caspase cleavage and accompanied by the maturity and release of pro-inflammatory mediators such as IL-1β and IL-18 (10, 11). The morphological characteristics, occurrence, and regulation mechanisms of pyroptosis are different from other types of cell death such as apoptosis and necrosis. Nucleotide-binding oligomerization domain like receptor (NLR) family proteins serve as sensors that recognize DAMPs and pathogen-associated molecular patterns (PAMPs), including high cytosolic Ca^2+^ with reduced K^+^ concentrations, extracellular ATP, mitochondrial dysfunction, and lysosomal rupture (10). These stimuli initiate caspase-dependent canonical or non-canonical inflammasome assembly (12), which subsequently cleaves gasdermin, a recently discovered pyroptosis effector. The N-terminal of gasdermin forms pores on the cell membrane, causing the release of mature inflammatory mediators into the extracellular matrix, and eventually leading to a severe inflammatory cascade reaction (13). Increasing evidence indicates that pyroptosis is induced in central nervous system disease including ischemic stroke (14, 15), traumatic brain injury (TBI) (16, 17), multiple sclerosis (MS) (18), Alzheimer's Disease (AD) (19), and Parkinson's disease (PD) (20). Illuminating the pyroptotic procedure would speed up the development of a cure for those diseases.

Gasdermin D (GSDMD), a 487 amino acid cytoplasmic protein, has been discovered to form membrane pore and act as a key effector for pyroptosis. N-terminal of GSDMD (GSDMD-N) is responsible for pore-forming activity, while the C-terminal domain (GSDMD-C) exerts autoinhibition on GSDMD-induced pyroptosis by binding to the N-terminal (21–23). Studies have confirmed that GSDMD participates in a series of pathologically pyroptotic events (21, 23), including ischemia/reperfusion (I/R) injury-induced pyroptosis. Experimental findings by Lee et al. showed that increased expression of NLRP3 inflammasome components and GSDMD peaked at 48 h after penetrating ballistic-like brain injury (24). Inhibition of caspase-1 mediated pyroptosis by limiting apoptosis-associated speck-like protein containing a CRAD (ASC) oligomerization and GSDMD cleavage resulted in suppressed expression of IL-1β and IL-18, and subsequent alleviation of BBB-disruption and brain injury (16, 17). Many studies have revealed the involvement of pyroptosis in cerebral injury (25, 26). However, the main cell type for pyroptosis, as well as the detailed information regarding GSDMD cleavage and its contribution to global inflammatory profile, still needs to be clarified. This study, utilizing *gsdmd*^−/−^ mice and *gsdmd*^−/−^ microglia cells, highlights the precise function of GSDMD, main pyroptotic effector cell, and canonical or non-canonical inflammasome-dependent pyroptosis pathway in cerebral I/R injury, aiming to elucidate the mechanisms underlying GSDMD-mediated pyroptosis and to exploit new therapeutic targets for ischemic stroke.

## Materials and Methods

### Animals and Stroke Model

Wild type C57BL/6 mice (*n* = 80, 8–10 weeks old, 20–25 g) were purchased from the Shanghai Laboratory Animal Center, Chinese Academy of Science (SLACCAS, Shanghai, China). All mice were housed in a standardized animal care center under a 12-h light-dark cycle with free access to food and water. All experimental operations were approved by the Ethics Committee of Wenzhou Medical University and were carried out in strict accordance with the animal care and use guidelines of the National Institutes of Health. GSDMD knockout (*gsdmd*^−/−^) mice (*n* = 20) were purchased from the Nanjing Biomedical Research Institute of Nanjing University (SCXK 2015-0001). All the *gsdmd*^−/−^ mice were generated from heterozygous breeding pairs of C57BL/6 mice.

The wild type C57BL/6 mice were randomized into a Sham group (*n* = 20), an MCAO model group (*n* = 40), and *gsdmd*^−/−^ group (*n* = 20). The middle cerebral artery occlusion (MCAO) model was established by intraluminal suture as described previously (27). Briefly, wild type C57BL/6 mice in the model group and the *gsdmd*^−/−^ mice inhaled 8% isoflurane. The inhalation anesthesia was maintained using a face mask with 4% isoflurane in a 5-l/min oxygen flow. The mice were then placed on a heating device to sustain a constant body temperature of 37°C. The right common, internal, and external carotid arteries (CCA, ICA, and ECA) were gently dissected and exposed. With a blockage of ICA at the proximal cranial end and the proximal end of the CCA, a suitable nylon thread was then inserted into the anterior cerebral artery to block the blood flow of the middle cerebral artery (MCA). After blockage for 1 h, the nylon thread was pulled out to allow reperfusion. The sham operated mice accepted the same operations, except for the occlusion of CCA. The mortality of the Sham group was around 5%. Mortality of the model group and *gsdmd*^−/−^ group was 13 and 10%, respectively at 24 h and reached 22 and 16% at 72 h.

### Cell Culture and OGD/R

BV2 microglia cells were purchased from iCell Bioscience Inc. (iCell-m011) and cultured in Dulbecco's minimal essential medium (DMEM) with 10% FBS (Gbico, USA), 100 U/ml penicillin, and 100 U/ml streptomycin under standard conditions. Oxygen and glucose deprivation/reperfusion (OGD/R) were conducted using an anaerobic incubator. Briefly, cells were incubated in a glucose-free DMEM (Thermo Fisher Scientific, USA) in a humidified 37°C anaerobic incubator supplied with a gas mixture of 94% N_2_, 5% CO_2_, and 1% O_2_ for 2-6 h. Reperfusion was allowed in normal conditions at 37°C for 12 h.

### Detection of Local Cerebral Blood Flow (CBF), Body Temperature, Blood Gas, and Blood Glucose

Mice were anesthetized by intraperitoneal injection of ketamine (90 mg/kg) and xylazine (10 mg/kg). The anesthetized mice were fixed on a stereotaxic apparatus and given local anesthesia on the top of the head with lidocaine. The skull was exposed by a median incision. Tissues adhering to the skull were removed by 3% H_2_O_2_. A probe was fixed at the core blood supply area of MCA (2.0 mm posterior to anterior fontanelle and 6.0 mm left from midline) to monitor local CBF. The MCAO model was established 10 min later. CBF value was recorded after 1 h of MCAO and 20 min of reperfusion. Taken the baseline value of CBF as 100%, the ratio of the CBF after MCAO/reperfusion to the baseline value represents the variation rate of CBF. The blood sample was taken from the submandibular vein of the mice. The body temperature, PH value of venous blood, oxygen partial pressure (PO_2_), carbon dioxide partial pressure (PCO_2_), and blood glucose of the mice were monitored 5 min before and after the operation. The CBF was monitored by a laser Doppler monitor (moorVMS-LDF, Wilmington, Delaware, USA). The body temperature of the mice was kept at 36 to 37°C by the 69001 temperature maintenance instrument (RWD Life Science Co., Ltd., Shenzhen, China). The blood gas of the venous blood was analyzed by the ABL80basic blood gas analyzer (Denmark Leidow). The blood glucose was measured by a One-touch blood glucose meter (Johnson & Johnson, US).

### TTC Staining

The brains of the mice were removed and cut into 2 mm sections 24 h after MCAO/reperfusion. The sections were then placed in 2% saline-dissolved 2,3,5-Triphenyltetrazolium chloride (TTC, sigma) for 30 min at room temperature. After washing with PBS, the sections were placed from the frontal pole and photographed. Ultimately, the infarct volume was calculated by Image J software.

### Neurobehavioral Training and Evaluation

The neurobehaviors of the mice were evaluated using a modified neurological severity score (mNSS) after training as previously described (28). Briefly, the neurological symptoms were scored at 9 a.m. on day 1, 7, 14, 21, and 28 after ischemia. A higher score indicates a more severe neurological dysfunction. All mice were pre-trained for 14 days before operation until their performances reached a steady state. The scoring was performed in strict accordance with double blinding principles. Scoring of the same mouse was performed with a time interval of more than 20 min.

### Immunohistochemical Analysis

Mice were anesthetized and perfused with normal saline (NS) and 4% paraformaldehyde. After that, the brain was rapidly taken out and placed in 4% paraformaldehyde for 2 days followed by paraffin embedding and sectioning. After continuous rehydration with 30% sucrose solution and washing with PBS, endogenous peroxidase activity in the tissue sections was blocked by 3% H_2_O_2_ for 25 min at room temperature. Antigen crosslinking was then conducted followed by two PBS washes. After being blocked in blocking solution (5% Goat Serum Albumin; Sigma) for 1 h, the sections were then incubated with the primary antibody against MAP2 (1:20, Proteintech) overnight. After two PBS washes and subsequent incubation with secondary antibody (ASS3403; Abgent, San Diego, CA, USA) for 30 min, the sections were then incubated with DAB (Solarbio, DA1010) till proper color appeared.

### Immunofluorescence Assays

Brain sections were fixed with 4% paraformaldehyde at room temperature (RT) for 30 min and then incubated in PBST (0.4% triton in PBS) containing 5% bovine serum albumin solution (BSA, Sigma-Aldrich) for 30 min at 37°C to rupture cell membrane and block non-specific staining. After that, incubation with the primary antibodies against cleaved caspase-1 (1:25, CST), cleaved caspase-11 (1:25, Abcam), MAP2 (1:50, Santa Cruz), and TMEM119 (1:200, Abcam) was then conducted at 4°C overnight followed by crosslink with the corresponding secondary antibody at RT for 1 h. After the unbound antibodies were washed off, the nuclei were then stained with DAPI (Abcam) and photographed with a fluorescence microscope (Leica). The data were analyzed from 3 randomly selected microscope fields using Image J software.

### Western Blot Analysis

Brain tissue was removed from euthanized mice and lysed in a RIPA buffer containing a protease inhibitor cocktail. After protein quantification with a bicinchoninic acid (BCA) protein assay kit (Thermo Fisher Scientific, USA), the sodium dodecyl sulfate polyacrylamide gel electrophoresis (SDS-PAGE) was then conducted. After the protein was transferred to a 0.22- or 0.45-μm polyvinylidene fluoride membrane (pre-activated with methanol; Milli-pore, Billerica, MA, USA), the membrane was blocked with 5% milk for 2 h, followed by incubation at 4°C for 24 h with primary antibodies against the following proteins: NLRP3 (1:1,000, CST), GSDMD (1:1,000, Abcam), caspase-1 (p20, 1:1,000, CST), caspase-11 (1:1,000, affinity), IL-1β (p17, 1:400, Proteintech), and IL-18 (1:500, Affinity). Glyceraldehyde 3-phosphate dehydrogenase (GAPDH; 1:1,000, CST), α-tubulin (1:1,000, CST), or β-actin (1:1,000, CST) was used as an internal reference. After that, the membrane was washed and the protein was incubated with the corresponding secondary antibody (1:5,000) at RT for 2 h. Image J software was used to analyze immunoreactive bands. The intensity of the targeted protein signal was compared to GAPDH or β-actin intensity.

### Enzyme-Linked Immunosorbent Assay (ELISA)

Cortical tissues of the brain taken from mice at 12, 24, 48, and 72 h were used for ELISA. The secretion levels of IL-1β and IL-18 in cortical tissues and cell culture medium were measured by ELISA kits (Elabscience Biotechnology, Wuhan, China) according to the manufacturer's instructions.

### Construction of Lentiviral Vectors for GSDMD Knockdown and Overexpression

The lentiviral vector pLent-U6-GFP-puro was applied for knockdown of GSDMD. Briefly, the shRNA-GSDMD oligonucleotides were designed and annealed through primers (N-shRNA: 5′- GATCCCACCGCAGCATGAAAGGCACCTTCACGAATGAAGGTGCCTTTCATGCTGCA−3′; C-shRNA: 5′- CGCGTAAAAGCAGCATGAAAGGCACCTTCATTCGTGAAGGTGCCTTTCATGCTGCG−3′). The lentiviral plasmid carrying shRNA-GSDMD fragment together with packaging plasmids were co-transfected into 293T cells for packaging. Virus-containing media were then collected, filtered, and concentrated for targeted suppression. For overexpression of GSDMD, the full length of GSDMD was amplified, enzyme digested, and conjoined with plasmid vector pCDH-CMV-MCS-EF1-Puro (Genepharma, Shanghai, China), followed by co-transfection into lentivirus. Microglia BV2 cells were infected with lenti-GSDMD. Western blotting was applied to assess the effectiveness of GSDMD knockdown or overexpression.

### RNA Extraction and PCR

Total RNA was isolated by TRIzol (Invitrogen) and cDNA was obtained from reverse transcription of the total RNA by RevertAid First Strand cDNA Synthesis Kit. PCR amplification assay was conducted with primers (GSDMD-gtF1: CGATGGAACGTAGTGCTGTG; GSDMD-gtR1: TCCTTCCCAACCTGCTGTTG).

### Statistical Analysis

All data analysis is based on at least three independent experiments. The data are present as mean ± standard deviation (SD). Data analysis at different time points was carried out using two-way analysis of variance (ANOVA) followed by least significant difference (LSD) *post-hoc* analysis. For other data comparisons, one-way ANOVA was used, followed by LSD *post-hoc* analysis or Student's *t*-tests. We considered a *p*-value lower than 0.05 as significant.

## Result

### Ischemic Reperfusion Stroke Induces GSDMD-Mediated Pyroptosis

**Figure 1:**
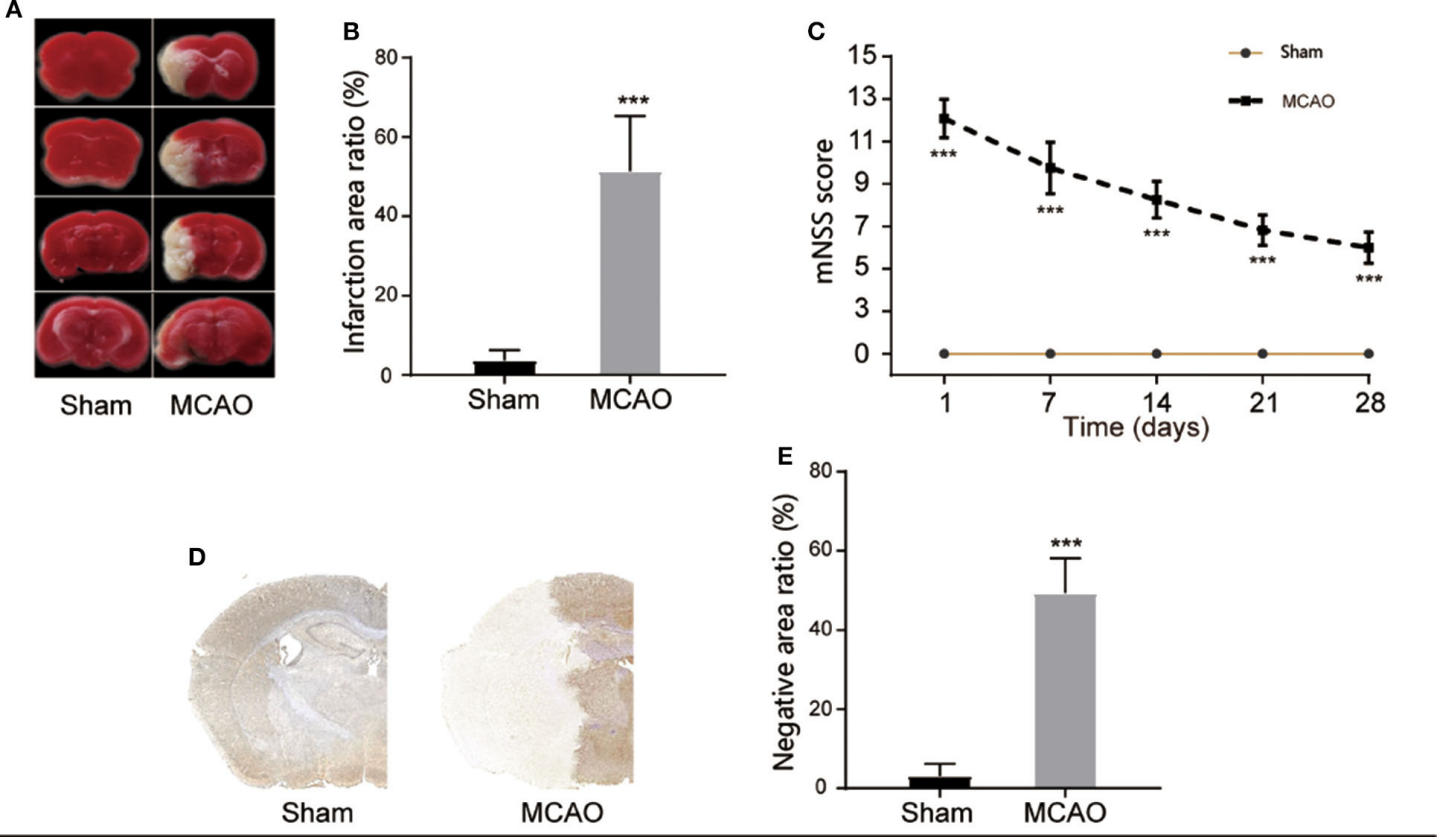
Construction of cerebral I/R model. **(A,B)** Representative images of TTC staining in four sequential brain slices from the mice in each group. *n* = 5. **(C)** Neurological recovery was evaluated by the mNSS scoring system on days 1, 7, 14, 21, and 28 post MCAO/R. *n* = 12. **(D,E)** Immunohistochemical staining of brain tissue at day 1 post MCAO/R. *n* = 5. ***P < 0.001, vs. the Sham group. I/R, ischemia reperfusion; MCAO, middle cerebral artery occlusion; mNSS, modified neurological severity score.

In order to study the pyroptosis in I/R, we firstly constructed the model of cerebral I/R in mice using the MCAO method. After the model had been establishment for 24 h, TTC staining showed that the subcortical tissue of the I/R ipsilateral brain was partially infarcted, and the infarcted area was approximately 51.35 ± 13.92% of the unilateral brain (Figures 1A,B). The neurological deficit score revealed that the MCAO group had a higher neurological deficit score (Figure 1C). The immunohistochemical staining also showed a significant infarcted area in the MCAO group (Figures 1D,E). These results indicate the successful construction of the cerebral I/R model in the mice.

**Figure 2:**
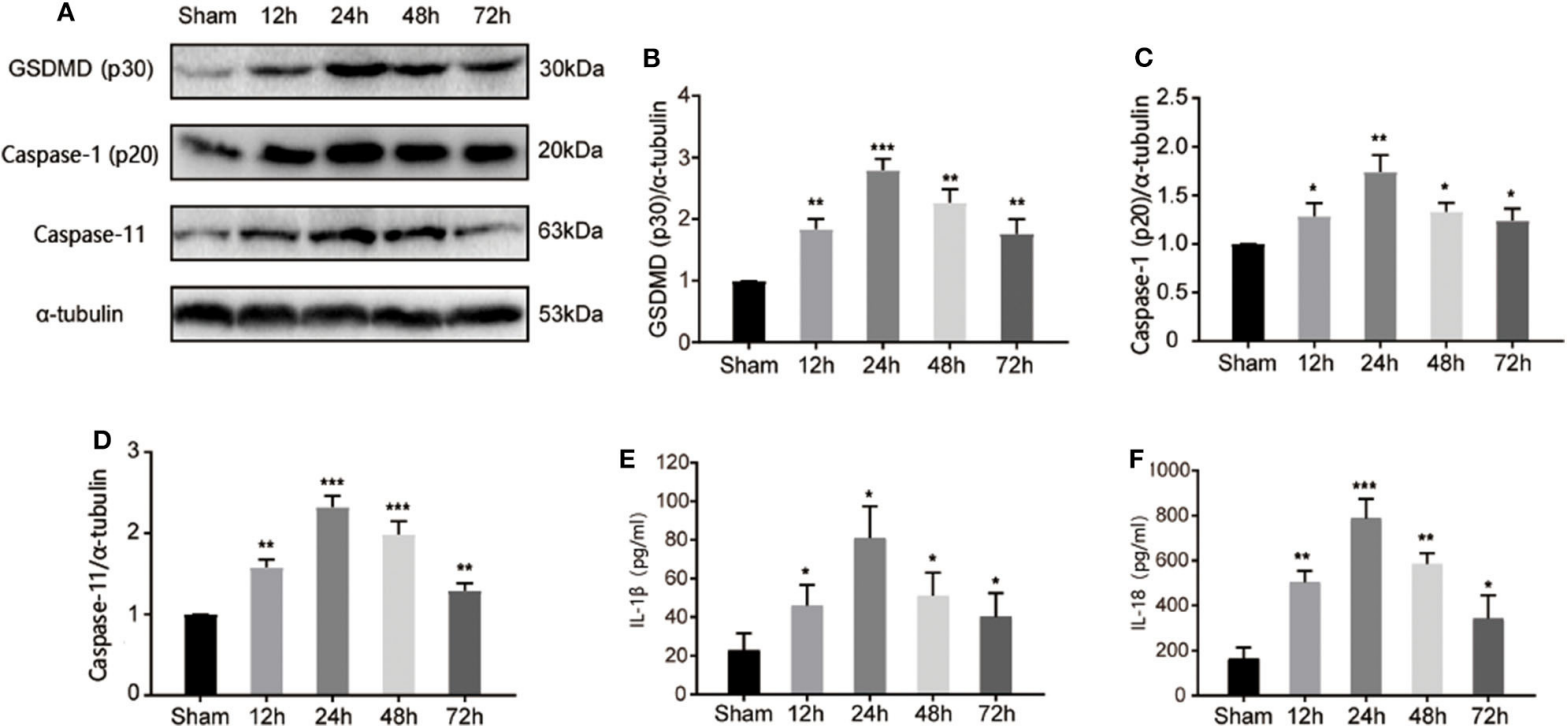
I/R injury induced canonical and non-canonical inflammasome dependent pyroptosis in ischemic cortex. **(A)** Band intensity of cleaved-GSDMD, active caspase-1, and caspase-11 at 12, 24, 48, 72 h post I/R injury. **(B–D)** Relative expression of cleaved-GSDMD **(B)**, active caspase-1 (p20) **(C)**, and caspase-11 **(D)** at 12, 24, 48, 72 h post I/R injury. *n* = 5. **(E,F)** The levels of IL-1β and IL-18 in the cortex were measured by ELISA at 12, 24, 48, 72 h post I/R injury. *n* = 5. *P < 0.05, **P < 0.01, ***P < 0.001, vs. the Sham group. I/R, ischemia reperfusion; GSDMD, gasdermin D.

GSDMD serves as a key response component in pyroptosis. To investigate the pyroptotic process during I/R injury, we detected the cleavage of GSDMD at different time points after the MCAO model was established. As shown in Figures 2A,B, cleaved GSDMD appeared at 12 h post I/R injury, and peaked at 24 h, indicating the initiation and development of GSDMD-mediated pyroptosis during I/R injury. As cleavage effectors of GSDMD, caspase-1, and caspase-11 regulate in canonical and non-canonical inflammasomes-dependent pathways. Through detecting the expression of active caspase-1 and caspase-11 by immunoblotting in the ischemic penumbra, we found that both caspase-1 and caspase-11 were activated and exhibited the same expression tendency as cleaved GSDMD (Figures 2C,D), which subsequently resulted in the production of mature IL-1β and IL-18 (Figures 2E,F). These results indicated that both canonical and non-canonical inflammasomes were involved in I/R induced pyroptosis.

### Pyroprosis Occurs Mainly in the Microglia

**Figure 3:**
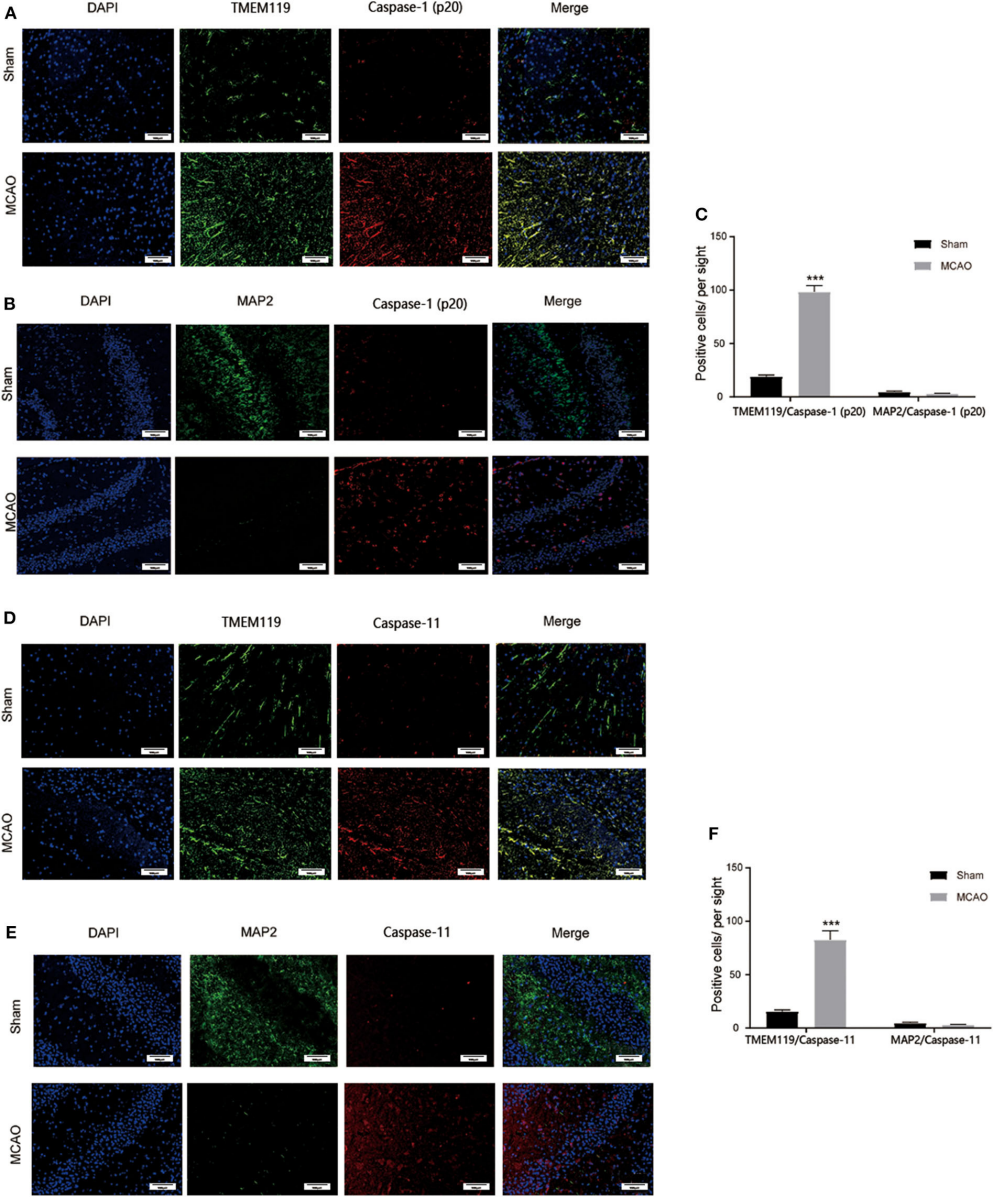
Main cell type for pyroptosis post I/R injury. **(A)** Caspase-1^+^ (red) and TMEM119^+^ (green) cells were significantly induced in the cortex at 24 h post I/R injury. **(B)** Caspase-1^+^ (red) cells were largely induced while MAP2^+^ (green) neurons were rarely detected in the cortex at 24 h post I/R injury. **(C)** Statistical analysis of immunofluorescence-positive cells. **(D)** Caspase-11^+^ (red) and TMEM119^+^ (green) cells were significantly induced in the cortex at 24 h post I/R injury. **(E)** Caspase-11^+^ (red) cells were largely induced while MAP2^+^ (green) stained neurons were rarely detected in the cortex at 24 h post I/R injury. **(F)** Statistical analysis of immunofluorescence-positive cells. Scale bars: 100 μm. *n* = 5. Data are presented as mean ± SD. ***P < 0.001, vs. the Sham group. I/R, ischemia reperfusion.

To further detect which type of cells in the brain were involved in I/R injury induced pyroptosis, double-immunofluorescence staining of MAP2 plus cleaved caspase-1, MAP2 plus cleaved caspase-11, TMEM119 pus cleaved caspase-1, and TMEM119 pus cleaved caspase-11 was applied to locate pyroptosis. Figure 3 shows that after brain I/R injury, MAP2^+^ cells were significantly reduced while TMEM119^+^ cells were increased significantly, indicating the death of neurons and activation of microglia cells post MCAO. We also found that cleaved caspase-1/11^+^ cells increased significantly and well-overlapped with TMEM119^+^ cells, whereas the surviving neurons displayed negligible positive staining of caspase-1 and caspase-11. Taken together, these results suggested that microglia might be the main effector cell to activate pyroptosis in I/R injury.

### GSDMD Promotes Pyroptosis in Microglia Cells

**Figure 4:**
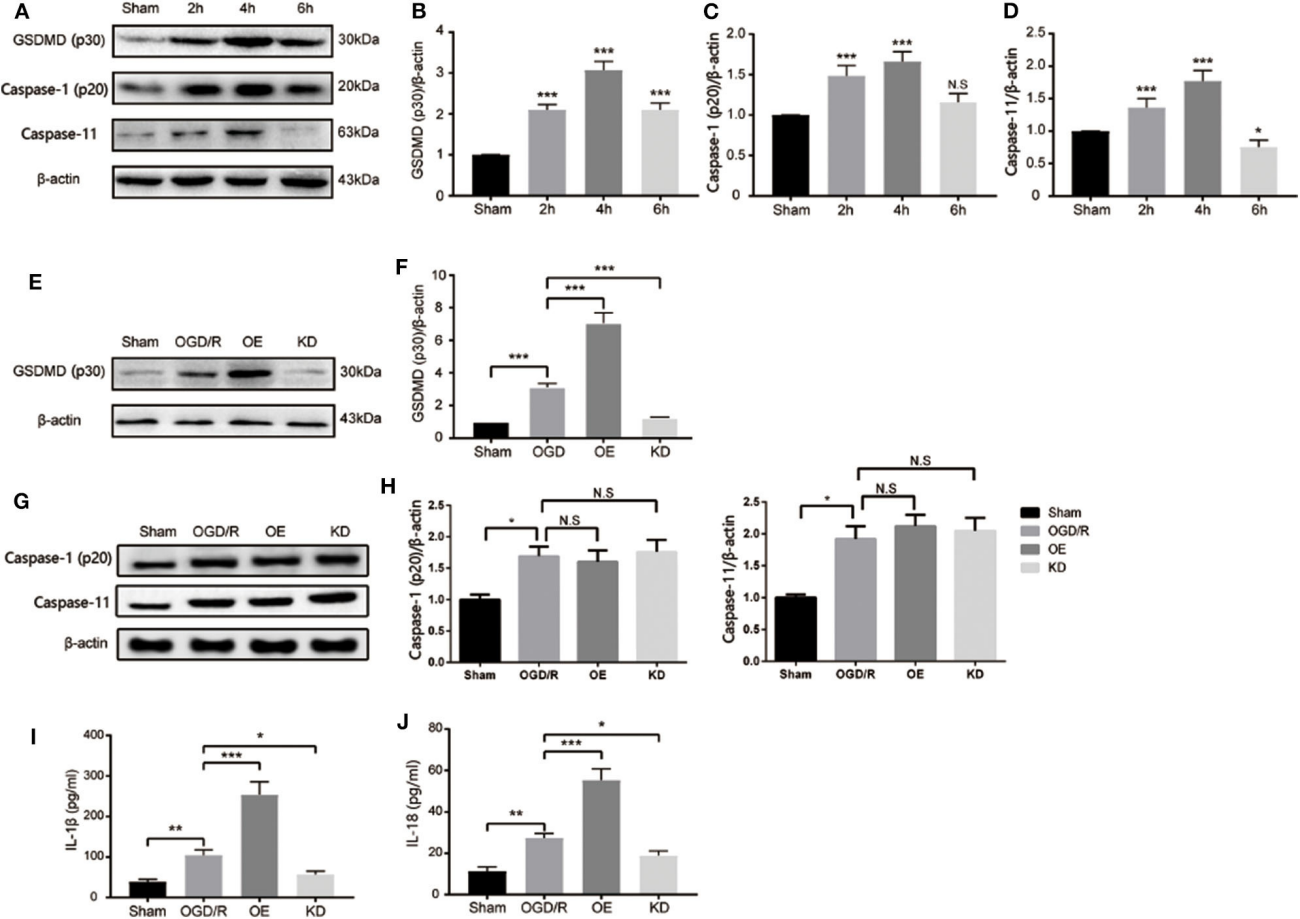
GSDMD is necessary for I/R induced pyroptosis and proinflammatory cytokine secretion in microglia BV2 cells. **(A–D)** Western blot analysis of the relative expression of cleaved-GSDMD **(B)**, active caspase-1 (p20) **(C)**, and caspase-11 **(D)** in microglia after OGD/R. **(E,F)** Relative expression of cleaved-GSDMD post shRNA-GSDMD interference or GSDMD overexpression in microglia. *n* = 5. **(G,H)** Relative expression of caspase-1 and caspase-11 in microglia post GSDMD silence or GSDMD overexpression. *n* = 5. **(I,J)** The expression of mature IL-1β and IL-18 in microglia BV2 cells were measured by ELISA at 4 h post OGD/R. *n* = 3. Data are presented as mean ± SD. *P < 0.05, **P < 0.01, ***P < 0.001 vs. the Sham group or relative group. GSDMD, gasdermin D; I/R, ischemia reperfusion; OGD, oxygen glucose deprivation.

After cerebral pyroptosis was confirmed in microglia post stroke induction, we next investigated the precise role of GSDMD in stroke-induced microglia. An OGD/R model of BV2 microglia was established to mimic I/R injury *in vitro*. As shown in Figures 4A,B, cleaved GSDMD (p30) was significantly elevated 2 h after OGD/R, and peaked at 4 h, indicating GSDMD-mediated pyroptosis was induced in microglia by OGD/R injury. However, BV2 microglia cells showed a high death rate after 6 h of OGD/R treatment, which might be attributed to the decreased expression of GSDMD. The expression of caspase-1 and caspase-11 was also detected and exhibited the same increasing tendency as GSDMD expression (Figures 4A,C,D). These results determined that GSDMD-mediated pyroptosis was activated in microglia by OGD/R injury.

To further clarify the mechanism of GSDMD in pyroptosis and related inflammatory responses in microglia, we detected the relevant factors after interfering with the expression of GSDMD. As shown in Figures 4E,F, the addition of exogenous GSDMD increased the content of GSDMD protein in microglia, while shRNA-GSDMD caused a decrease in GSDMD-p30 protein content 4 h after OGD/R injury. Neither exogenous GSDMD nor shRNA-GSDMD influenced the expression of caspase-1 and caspase-11 in microglia (Figures 4G,H; *P* > 0.05). The secretion changes of IL-1β and IL-18 were measured by ELISA. OGD/R treatment increased the media secretion of IL-1β and IL-18 from 38.81 ± 6.311 pg/ml, 11.25 ± 2.125 pg/ml to 104.9 ± 12.92 pg/ml, and 27.48 ± 2.104 pg/ml, respectively. Overexpression of GSDMD significantly promoted the secretion levels of IL-1β and IL-18 to approximately 254 ± 31.88 and 55.3 ± 5.397 pg/ml (Figures 4I,J). However, the absence of GSDMD attenuated the increased secretion levels of IL-1β and IL-18 (Figures 4I,J). Taken together, GSDMD mediated pyroptosis-dependent inflammation reaction in microglia.

### Knockout of GSDMD Accelerates Neurological Recovery From I/R Injury

**Figure 5:**
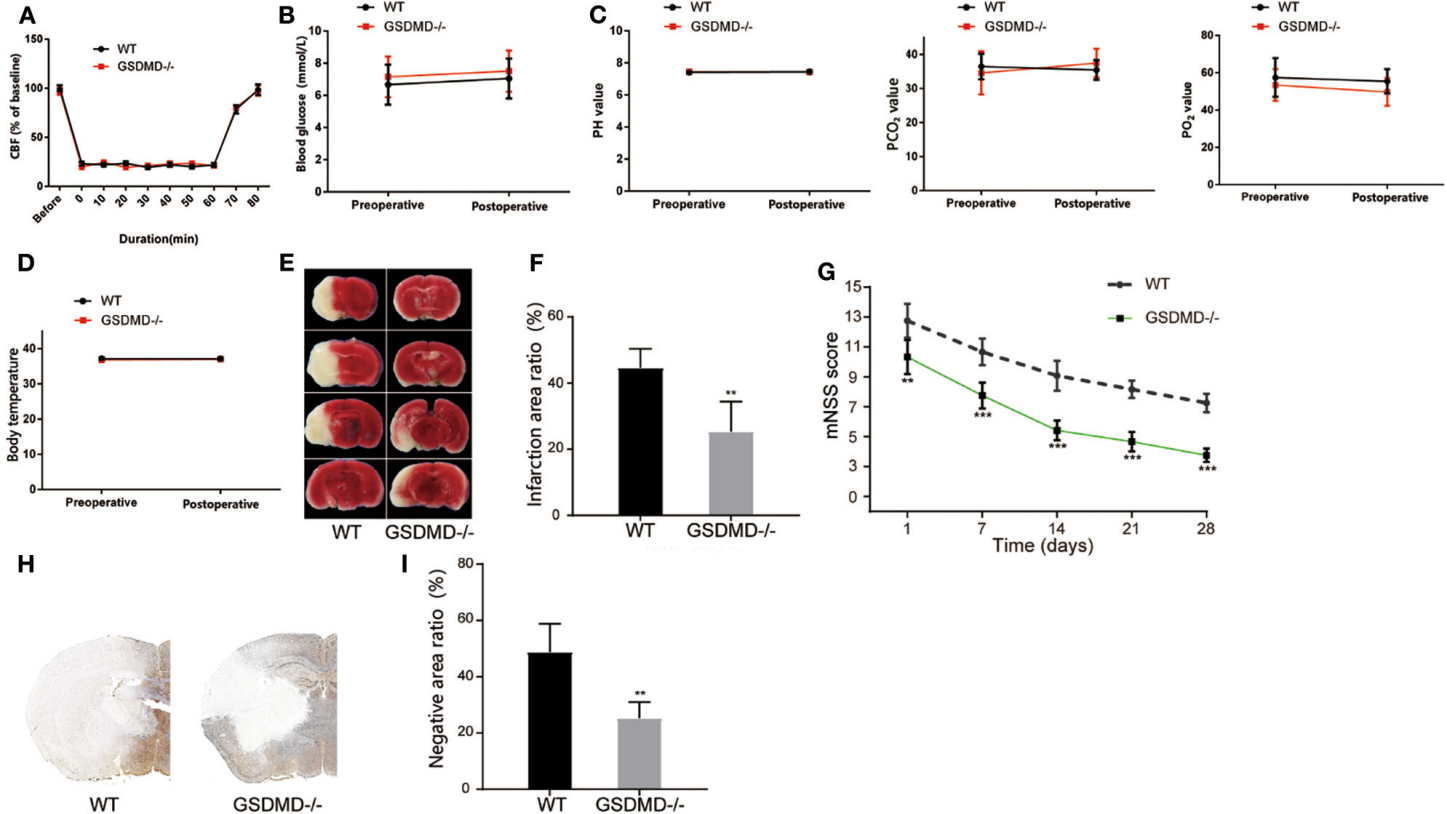
Ablation of GSDMD improves neurological outcome post I/R injury. **(A)** CBF in the MCAO modeling. *n* = 5. **(B–D)** Blood glucose **(B)**, blood gas **(C)**, and body temperature **(D)** of the mice before and after MCAO modeling. *n* = 5. **(E,F)** Representative images of TTC staining in four sequential brain slices from WT and *gsdmd*^−/−^ mice. *n* = 5. **(G)** Neurological recovery was evaluated by mNSS on days 1, 7, 14, 21, and 28 post MCAO/R. *n* = 12. **(H,I)** Immunohistochemical staining of brain tissue on day 1 post MCAO/R. *n* = 5. **P < 0.01, ***P < 0.001, vs. the WT group. GSDMD, gasdermin D; I/R, ischemia reperfusion; MCAO, middle cerebral artery occlusion; CBF, cerebral blood flow; mNSS, modified neurological severity score; WT, wild type.

To further validate the effective role of GSDMD protein in I/R induced pyroptosis *in vivo*, we constructed *gsdmd*^−/−^ C57BL/6 mice by CRISPR-Cas9 technology. Firstly, the cerebral I/R model was established in wide type (WT) and *gsdmd*^−/−^ C57BL/6 mice. The local CBF was monitored according to the technical standards of the Stroke Treatment Academic Industry Roundtable (STAIR). The local CBF of WT mice dropped from 100% to about 20% after MCAO, while returned to 100% after the thread plug was removed. The CBF of *gsdmd*^−/−^ mice which were treated in the same way as the WT mice had similar variations (Figure 5A, *P* > 0.05). The body temperature, blood gas, and blood glucose of WT and *gsdmd*^−/−^ mice were also measured before and after modeling and showed no significant differences in these two groups of mice (Figures 5B–D; *P* > 0.05). Therefore, it was appropriate to compare the groups in the following experiments. TTC staining showed that 24 h after I/R, the cerebral infarction of the *gsdmd*^−/−^ group reduced to 25.37 ± 9.074% compared to 44.55 ± 5.834% in the WT group (Figures 5E,F; *P* < 0.01). Twenty-eight days after stroke, the mNSS scores of the *gsdmd*^−/−^ group and WT group were 3.75 ± 0.4523 and 6.25 ± 0.6216, respectively (Figure 5G, *P* < 0.001), indicating that the absence of GSDMD significantly promoted neurological recovery from I/R injury. The result of immunohistochemical staining was consistent with that of the TTC staining (Figures 5H,I), suggesting that the absence of GSDMD effectively reduced the infarct volume.

### Knockout of GSDMD Prevents Caspase-1 and Caspase-11 Mediated Inflammatory Response

**Figure 6:**
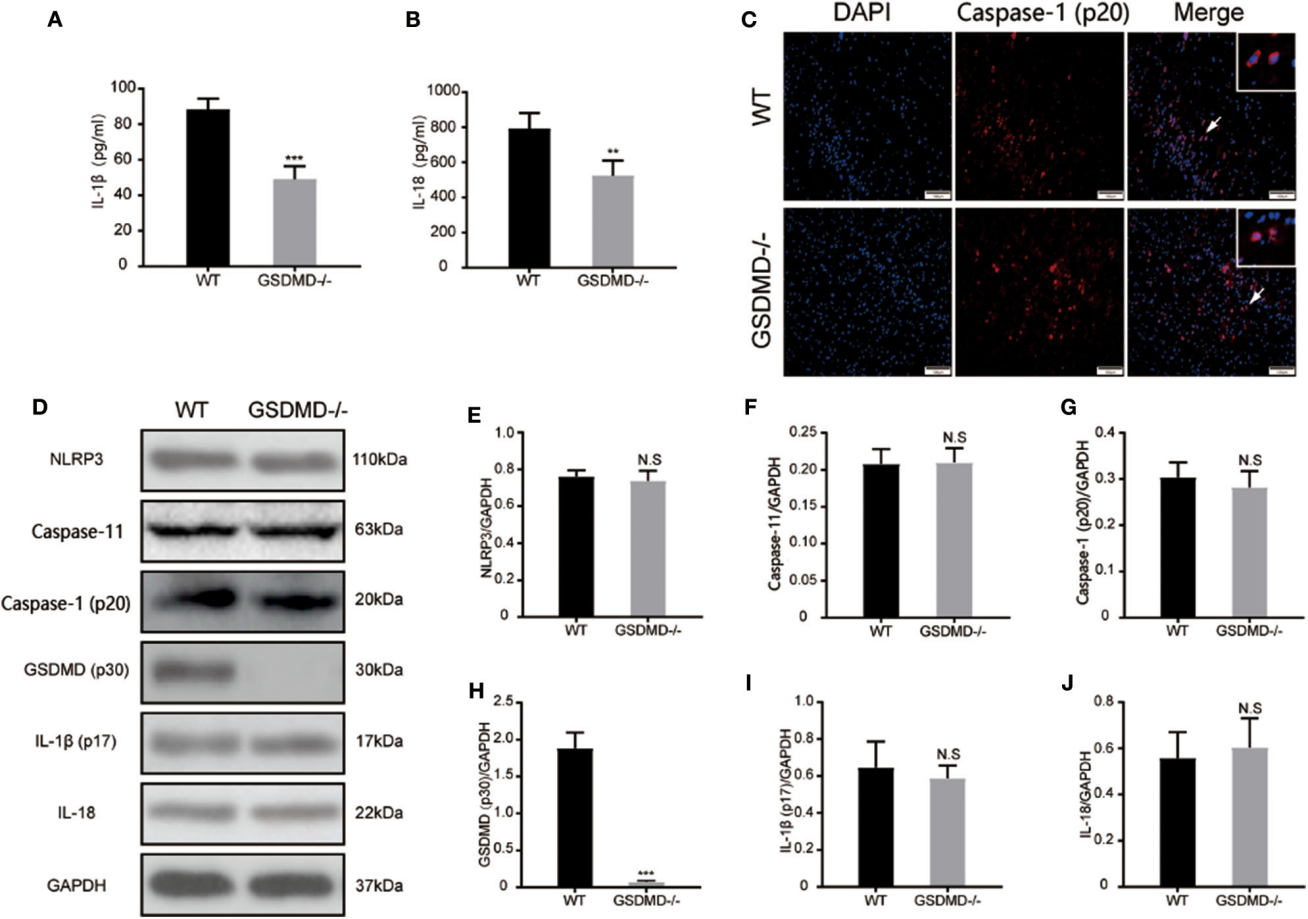
Ablation of GSDMD reduces pyroptosis and global neuroinflammation post I/R injury. **(A,B)** Secretion of mature IL-1β and IL-18 into plasma was measured by ELISA at 24 h post I/R injury. *n* = 5. **(C)** Representative photographs of immunofluorescence staining for cleaved caspase-1 (red) in the cortex at 24 h post-injury. *n* = 5. **(D–J)** Western blotting assay and statistical analysis of the relative expression of NLRP3 **(E)**, caspase-11 **(F)**, active caspase-1**(G)**, cleaved GSDMD **(H)**, active mature IL-1β **(I)**, and IL-18 **(J)**. *n* = 5. **P < 0.01, ***P < 0.001 vs. the WT group. GSDMD, gasdermin D; I/R, ischemia reperfusion; WT, wild type.

To further validate the role of GSDMD protein in the global inflammatory response during I/R injury, the secretion of IL-1β and IL-18 was detected through ELISA. As shown in Figures 6A,B, the expressions of plasma IL-1β and IL-18 were significantly attenuated in the *gsdmd*^−/−^ group, approximately from 88.35 ± 5.993 pg/ml and 793.3 ± 87.35 pg/ml to 49.12 ± 7.224 pg/ml and 524.8 ± 86.33 pg/ml, respectively. The immunofluorescence staining of cleaved caspase-1 (Figure 6C) showed that blockage of GSDMD did not affect the expression or cleavage of caspase-1, suggesting that the knockout of GSDMD had no influence on the upstream process of pyroptosis but that it significantly blocked the release of inflammatory cytokines. Furthermore, through detecting caspase-1 and caspase-11 mediated pyroptosis pathways post I/R injury, the expression of caspase-1 (p20), caspase-11, mature IL-1β, IL-18, and NLRP3 proteins in the ischemic cortex showed no significant changes in the absence of GSDMD (Figures 6D–J). These results suggested that knockout of the GSDMD gene blocked the release of mature IL-1β and IL-18, but did not affect the process of maturation, which was consistent with other studies (13).

## Discussion

In this study, we demonstrated that cerebral pyroptosis was significantly induced around the infarcted area in the acute I/R injury. Our results revealed that knockdown of GSDMD exhibited promising therapeutic effects, including improving neural function, reducing cortical lesion volume, and attenuating global inflammation *in vitro* and *in vivo*. In addition, we confirmed that microglia might be the main pyroptotic effector cell in the ischemic brain. Moreover, the ablation of GSDMD could effectively reduce the secretion of mature IL-1β and IL18. Brain damage following I/R injury is a complicated pathophysiological problem, accompanied by a variety of interrelated cellular and molecular changes. These changes include neuronal apoptosis, BBB disruption, mitochondrial dysregulation, reactive oxygen species (ROS) accumulation, and inflammatory reaction (25, 29). Overall, cerebral ischemic injury causes a large number of irreversible neuron deaths. Although timely reperfusion prevents further neuron deaths, the ongoing reperfusion triggers secondary brain injuries, including neural inflammation, oxidative damage, cell apoptosis, and necroptosis, which finally leads to cerebral infarct and neurological deficit (30, 31). In this study, WT mice exhibited a significant neurological deficit and plentiful nerve cell deaths after I/R, while *gsdmd*^−/−^ mice gained a neural recovery, suggesting GSDMD acted as a crucial protein in I/R induced secondary injury.

Pyroptosis has been identified as a programmed cell necroptosis associated with inflammatory and antimicrobial responses. Pyroptosis is featured by cell membrane perforation and disintegration, which eventually result in cell lysis (32). The initiation of pyroptosis is caused by recognition of PAMPS and DAMPs through NLRand Toll-like receptor (TLR) family proteins which trigger caspase-dependent cleavage of GSDMD. GSDMD-N binds to lipids in the plasma membrane and forms large oligomeric pores with a diameter of 10–20 nm, causing the release of pro-inflammatory mediators IL-1β and IL-18 (33, 34). Therefore, preventing GSDMD cleavage and membrane pore formation might terminate cell pyroptosis. To date, caspase-1 dependent canonical inflammasomes and caspase-4/5/11 dependent non-canonical inflammasomes are found to be involved in the cleavage of the GSDM family (34, 35). In our study, canonical inflammasome and non-canonical inflammasome mediated pyroptosis was detected during I/R injury, evidenced by increased expression of NLRP3, caspase-1/11, GSDMD, and IL-1β/18 (Supplementary Figure 1). Further investigations into the process of inflammasome assembly may provide new insights into I/R injury induced pyroptosis.

Neuroinflammation is considered as the main cause of secondary brain injury during I/R. Immune cells recognize certain danger signals which boost production and the release of cytokines like TNF-α, IL-1β, IL-6, MCP-1, and MIP-1α, leading to activation of inflammatory pathways and neural damage (36, 37). As a pro-inflammatory process, pyroptosis causes not only rapid loss of membrane integrity but also the release of mature IL-1β and IL-18, which induces the strong pro-inflammatory activity of immune cells. A pore of about 20 nm was created in the cell membrane, allowing for water influx and leading to cell swelling and cellular breakdown (38). In our study, through interrupting I/R injury induced pyroptosis by inhibition of GSDMD, we successfully reduced expression of IL-1β and IL-18 in plasma but not in ischemic tissue, suggesting that GSDMD pore formation contributed to the release of proinflammatory cytokines from immune cells. Since GSDMD activity can influence the secretion of mature IL-1β and IL-18, further investigation could be made into the potential of GSDMD inhibition for inflammation expansion and neuron protection.

Consistent with our study, Zhang et al. have identified GSDMD as a key executioner in caspase-1-mediated pyroptosis during I/R injury (26). However, the cell candidate for I/R induced pyroptosis remains unclear. As the main immune cells resident in brain tissue, it has been proposed that microglia play an essential role in the progression of neuroinflammation (39). A recent study demonstrated that microglia acquired an M1 phenotype and secreted abundant pro-inflammatory cytokines during I/R injury (40). Immunofluorescence assay in this study showed that the microglia was activated in I/R injury induced cerebral pyroptosis. Aggregated intracellular proinflammatory mediators are released into the extracellular matrix during microglia pyroptosis, causing neuronal inflammation around the ischemic penumbra (41, 42). As the inflammation continues, necrosis of the ischemia penumbra is aggravated. Based on *in vivo* and *in vitro* experiments, GSDMD knockout resulted in less pyroptotic microglia cells and attenuated global cerebral neuroinflammation, which was caused by blockage of pore formation in the cell membrane and reduction of the subsequent secretion of proinflammatory mediators. We also found massive neuron deaths in the ischemic penumbra even though GSDMD was inhibited, suggesting that ischemia induced neuron death was irreversible. Therefore, rescuing neurons nearby the ischemic penumbra is still of great importance.

There are some limitations to this study. Firstly, apart from NLRP3-driven inflammasome assembly, we did not dig into other NLRs or TLRs which also trigger the initiation of pyroptosis during I/R injury. Secondly, we only performed immunofluorescence of GSDMD and cleaved caspase-1 in neurons and microglia to find out that ischemic stroke induced pyroptosis mainly occurs in microglia that resides in the brain tissue, but did not exclude the possibility of other immune cells like astrocytes, neutrophils, and permeated macrophages being the candidate for stroke induced pyroptosis. Despite these limitations, this study provides new insights into neurological recovery and stroke treatment. Since pyroptosis is not responsible for neuron death during ischemic stroke, the rescue of neurons may be of great significance to neurological recovery. Other forms of cell death, including apoptosis, necrosis, and ferroptosis, may also play certain roles in secondary cerebral injury. Future research may focus on immune cell death during cerebral I/R injury.

## Conclusion

**Figure 7:**
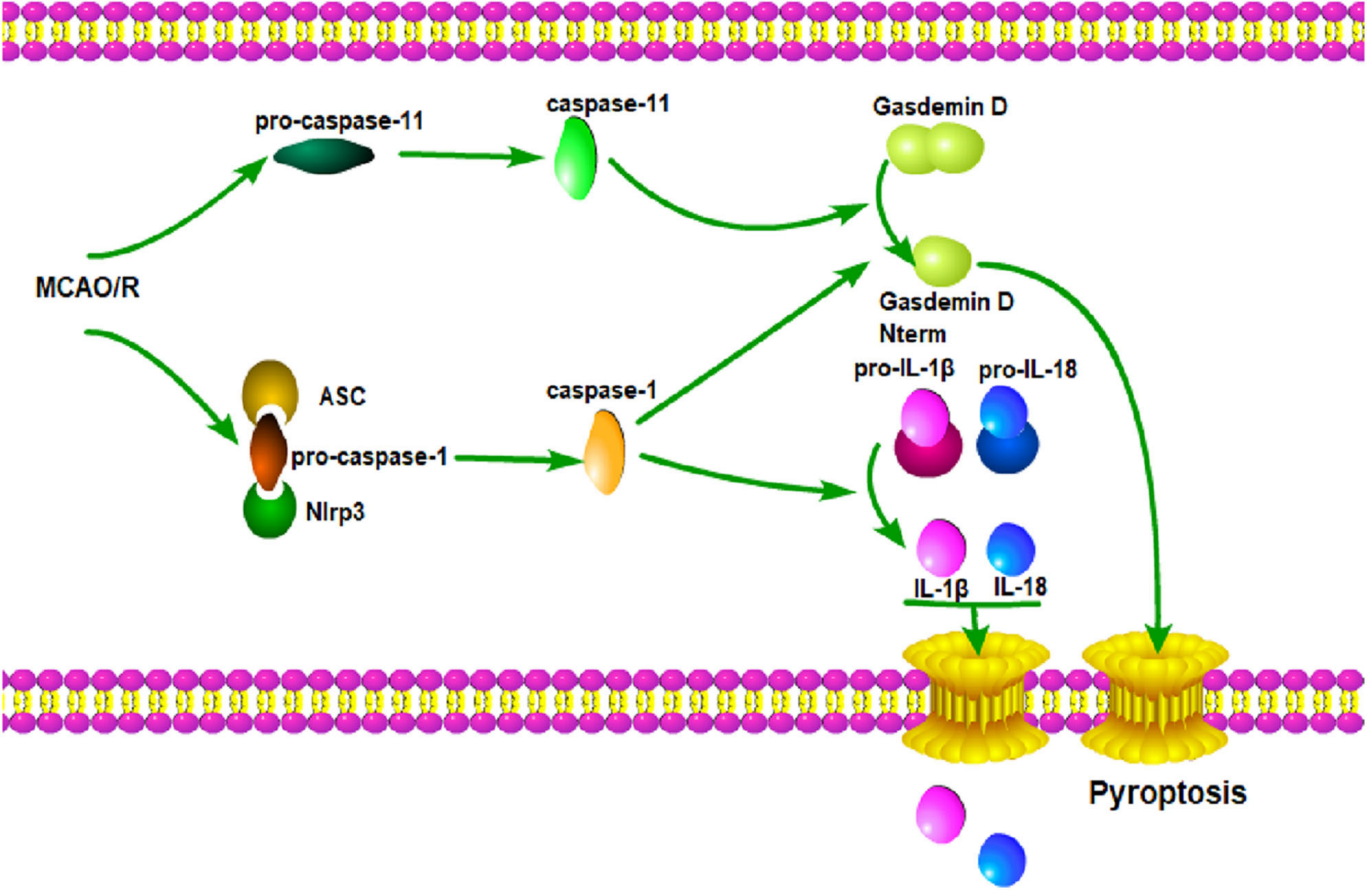
GSDMD mediated pyroptosis pathways. Cerebral I/R damage induces the activation of caspase-1 and caspase-11; caspase-1 leads to the maturation of IL-1β and IL-18; caspase-1 and caspase-11 induce the cleavage of GSDMD and release of GSDMD-N; GSDMD-N forms a large pore in the plasma membrane; IL-1β and IL-18 are released through the pore; membrane integrity is disrupted, which ultimately leads to pyroptosis.

In summary, our results indicate that the knockout of GSDMD exerts a neuroprotective effect by inhibiting microglia pyroptosis and global neuroinflammation in mice with I/R injury. Therefore, a miRNA modulator and/or genetic regulator targeting GSDMD might serve as a promising alternative molecular drug for the treatment of ischemic stroke. Here, a diagram depicting the working mode of microglia pyroptosis signaling after cerebral I/R is supplemented (Figure 7).

## Data Availability Statement

All datasets generated for this study are included in the article/Supplementary Material.

## Ethics Statement

The animal study was reviewed and approved by the Ethics Committee of Wenzhou Medical University.

## Author Contributions

SY, LH, and QZ designed the experiments and edited the manuscript. KJ gave guidance to the experiments. KW, ZS, JR, and XL performed the experiments and analyzed the data. KW, SW, and LR interpreted the data and prepared the figures. SY and KW wrote the manuscript. All authors contributed to the article and approved the submitted version.

## Conflict of Interest

The authors declare that the research was conducted in the absence of any commercial or financial relationships that could be construed as a potential conflict of interest.
