# Supplementary figures and images for: Ablation of GSDMD Improves Outcome of Ischemic Stroke Through Blocking Canonical and Non-canonical Inflammasomes Dependent Pyroptosis in Microglia
Source: Front Neurol. 2020 Nov 23;11:577927. doi: 10.3389/fneur.2020.577927 (PMC7719685; doi:10.3389/fneur.2020.577927)

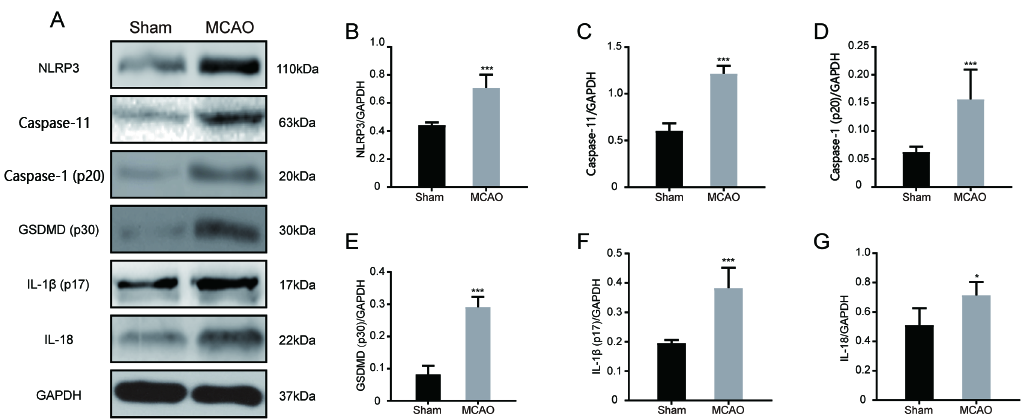

Supplement: Supplementary file 1 [file Image_1.TIF]
